# NMR and MD Studies Reveal That the Isolated Dengue NS3 Protease Is an Intrinsically Disordered Chymotrypsin Fold Which Absolutely Requests NS2B for Correct Folding and Functional Dynamics

**DOI:** 10.1371/journal.pone.0134823

**Published:** 2015-08-10

**Authors:** Garvita Gupta, Liangzhong Lim, Jianxing Song

**Affiliations:** Department of Biological Sciences, Faculty of Science, National University of Singapore, 10 Kent Ridge Crescent, Singapore, Singapore; nanyang technological university, SINGAPORE

## Abstract

Dengue genome encodes a two component protease complex (NS2B-NS3pro) essential for the viral maturation/infectivity, thus representing a key drug target. Previously, due to its “complete insolubility”, the isolated NS3pro could not be experimentally studied and it remains elusive what structure it adopts without NS2B and why NS2B is indispensable. Here as facilitated by our previous discovery, the isolated NS3pro has been surprisingly deciphered by NMR to be the first intrinsically-disordered chymotrypsin-like fold, which exists in a loosely-packed state with non-native long-range interactions as revealed by paramagnetic relaxation enhancement (PRE). The disordered NS3pro appears to be needed for binding a human host factor to trigger the membrane remodeling. Moreover, we have *in vitro* refolded the NS3pro in complex with either NS2B (48–100) or the full-length NS2B (1–130) anchored into the LMPC micelle, and the two complexes have similar activities but different dynamics. We also performed molecular dynamics (MD) simulations and the results revealed that NS2B shows the highest structural fluctuations in the complex, thus providing the dynamic basis for the observation on its conformational exchange between open and closed states. Remarkably, the NS2B cofactor plays a central role in maintaining the correlated motion network required for the catalysis as we previously decoded for the SARS 3CL protease. Indeed, a truncated NS2B (48–100;Δ77–84) with the flexible loop deleted is able to trap the NS2B-NS3pro complex in a highly dynamic and catalytically-impotent state. Taken together, our study implies potential strategies to perturb the NS2B-NS3pro interface for design of inhibitors for treating dengue infection.

## Introduction

Dengue is the most prevalent mosquito-borne viral disease with over 500 million human infections annually and 2.5 billion people at risk, particularly in tropical and subtropical regions [1,2]. The disease is caused by Dengue virus (DENV) belonging to the *Flaviviridae* family, which also includes several other human pathogens such as the West Nile virus, Japanese encephalitis virus, and yellow fever virus [1–4]. So far, four antigenically distinct DENV serotypes have been identified: DENV-1 to -4, all of which cause dengue fever, dengue haemorrhagic fever and dengue shock syndrome. Despite intense studies, currently there are neither vaccines nor other treatments available to treat this disease [5,6].

The DENV genome is composed of an 11-kb single-stranded positive sense RNA. Upon infection, the RNA genome is translated into a large polyprotein by the host-cell translation machinery, that is subsequently processed into 10 proteins, including three structural proteins (capsid, membrane, and envelope) and seven nonstructural proteins (NS1, NS2A/B, NS3, NS4A/B, and NS5). The structural proteins form the viral particle while the nonstructural proteins participate in the replication of the RNA genome, virion assembly, and attenuation of the host antiviral response. The cleavage of the polyprotein is carried out by host cell proteases including furin and signalaseas, as well as a virus-encoded serine protease NS2B-NS3pro, which has been established as a valuable target of therapeutic interest [5,6]. The protease domain NS3pro consisting of the N-terminal part of NS3 adopts a chymotrypsin-like fold with two β-barrels, each composed of six β-strands, with the catalytic triad (His51-Asp75-Ser135) located at the cleft between the two β-barrels [7,8]. Amazingly, unlike other proteases with a chymotrypsin-like fold, the flavivirus proteases including dengue protease, requires a stretch of approximately 40 amino acids from the cytosolic domain of NS2B for catalytic activity, thus called two-component protease [6–8]. Intriguingly, while the protease domains adopt highly similar structures in all crystal structures determined to date, the NS2B cofactors have been found to assume two distinctive structures, namely open (inactive) and closed (active) conformations by X-ray crystallography and NMR spectroscopy [7–12].

To understand why the NS2B cofactor is indispensable for activating the dengue NS3 protease is not only of fundamental interest for enzymology, but also bears considerable implications for design of molecules with high affinity and specificity to inhibit the protease [13,14]. However, due to its “complete insolubility”, it has been previously impossible to carry out any experimental studies on the isolated NS3pro domain. In 2005, we discovered that previously-thought “insoluble proteins” in fact could be solubilized in water with minimized salt ions [15–18], and therefore we have used this to study various previously-thought insoluble proteins including TDP-43 N-terminus [19]. Here with this approach, we have successfully characterized the solution conformations and dynamics of the isolated protease domain by CD, NMR and paramagnetic relaxation enhancement (PRE). The results reveal that surprisingly the isolated NS3pro domain with the native sequence is intrinsically disordered without any stable secondary and tertiary structures, as well as has no detectable activity. Nevertheless, upon meeting the NS2B cofactor, the disordered NS3pro spontaneously folds into the well-structured and active enzyme highly similar to those co-expressed [10,11]. We have also successfully refolded the NS3pro complexed with the full-length NS2B anchored into the LMPC micelle. To further decipher the roles of the NS2B cofactor in protein dynamics, we performed molecular dynamics (MD) simulations which is very powerful in pinpointing the role of protein dynamics in the catalysis of proteases such as the SARS 3C-like protease [20,21]. The correlation analysis reveals that the NS2B residues play a central role in coordinating the correlated motion network in the NS2B-NS3pro complex. Indeed, a truncated NS2B is able to form a buffer-soluble complex with NS3pro, but this complex is highly dynamic and catalytically-impotent. Our results imply that the discovery/design of molecules to block the correct folding of the NS2B-NS3pro complex or/and to decouple its correlated motion network might represent promising strategies to inhibit the dengue protease, thus holding the considerable potential to treat dengue infections.

## Materials and Methods

### Plasmid construction

DNA sequences encoding the NS3 protease and NS2B from Dengue virus type 2 strain TSV01 (GenBank accession number AY037116) were optimized and synthesized by GenScript (Piscataway, NJ). With designed primers, the optimized DNA by GenScript were used as templates for amplifying the DNA fragments encoding residues 14–185 of NS3pro and residues 48–100 of NS2B, which have the same starting and ending residues as the constructs used in the previous NMR study [10], as well as the full-length NS2B over residues 1–130 including the N- and C-terminal transmembrane domains. Amplified DNA fragments for NS3 (14–185), NS2B (1–130) and were subsequently cloned into pET28a vector with N-terminal His-tag (Novagen) using NcoI and XhoI restriction sites, while fragments for NS2B (48–100) and NS2B (48–100;Δ77–84) with Thr77-Met84 replaced by three Gly residues were cloned into pGEX-4T1 vector with GST-tag (GE healthcare) using BamHI and XhoI restriction sites. DNA sequences of cloned constructs were verified by automated DNA sequencing.

### Protein expression and purification

Competent *Escherichia coli* BL21 (DE3) Star cells were transformed with pET28a-NS3pro or pGEX-4T1-NS2B plasmids. Transformed single colony was inoculated overnight in 10 ml Luria-Bertani broth containing 25 μg/ml kanamycin for pET28a and 100 μg/ml ampicillin for pGEX-4T1. Then cells were transferred to 1 lit LB media containing respective antibiotics and grown at 37°C with shaking until the A_600_ reached to 0.6. Cultures were induced with 0.5 mM isopropyl *β*-D-thiogalactopyranoside (IPTG) for 16 h at 20°C. Cells were harvested and resuspended in cold Phosphate Buffered Saline (PBS) pH 7.4 buffer for lysis by sonication. After centrifugation at 40000*g*, GST-tagged NS2B (48–100) protein was purified from supernatant using affinity chromatography. The GST-NS2B (48–100) protein was cleaved with thrombin to remove the GST tag, and the released NS2B peptide was further purified by RP-HPLC on a Vydac C_8_ column. The NS2B (1–130) and NS3pro proteins were completely insoluble and all found in inclusion body, which were solubilized with PBS buffer (pH7.4) containing 8 M urea. Cell debris was removed by centrifugation at 40000*g*, and supernatant containing His-tagged NS2B (1–130) and NS3pro were purified by Ni-NTA affinity chromatography under denaturing condition. Eluted protein was further purified with RP-HPLC on a Vydac C_8_ column.

(^15^NH_4_)_2_SO_4_, [^13^C6]-glucose and D_2_O were purchased from Cambridge Isotope Laboratories (Andover, MA). The generation of the isotope-labeled proteins for NMR studies followed a similar procedure except that the bacteria were grown in M9 medium with the addition of (^15^NH_4_)_2_SO_4_ for ^15^N labeling and (^15^NH_4_)_2_SO_4_/[^13^C]-glucose for ^15^N-/^13^C-double labelling as previously described [22]. The purity of the recombinant proteins was checked by SDS-PAGE gels and their molecular weights were verified by ESI-MS and Voyager STR matrix-assisted laser desorption ionization time-of-flight-mass spectrometer (Applied Biosystems). The concentration of protein samples was determined by the UV spectroscopic method in the presence of 8 M urea [23].

### Enzymatic Activity and Kinetics

The Dengue protease substrate peptide Bz-Nle-Lys-Arg-Arg-AMC was purchased from GenScript (Piscataway, NJ). Enzymatic activity of the isolated NS3pro and refolded NS2B-NS3pro was measured in the assay buffer containing 50 mM Tris-HCl (pH 7.5), 0.001% Triton X-100, 0.5 mM EGTA, or 100 mM sodium acetate (pH 4.0), 0.001% Triton X-100, 0.5 mM EGTA. Briefly, in the 100 μl reaction mixtures containing 0.3 μM protease, 20 μM protease specific fluorophore-tagged substrate benzoyl-Nle-Lys-Arg-Arg-aminomethylcoumarin (Bz-nKRR-AMC) was added and reaction mixtures were incubated at 37°C, and the liberated coumarin fluorophore was continuously monitored at λ_ex_ of 380 nm and λ_em_ of 450 nm on Infinite M200 PRO Tecan microplate reader. For steady state kinetics, 0.3 μM refolded NS3-NS2B protease was incubated with various concentrations of Bz-nKRR-AMC substrate in assay buffer containing 50 mM Tris-HCl (pH 7.5), 0.001% Triton X-100, 0.5 mM EGTA at 37°C. Progression of enzymatic reaction was monitored as an increase in fluorescence at λ_ex_ of 380 nm and λ_em_ of 450 nm. Initial fluorescence velocities (relative fluorescence units/sec) were calculated and curves were fitted to the Michaelis-Menten equation by nonlinear regression using GraphPad Prism. Steady-state kinetic constants were determined from triplicate measurements and reported as mean Standard Error.

### Site-directed mutagenesis and spin-labeling

As the NS3pro contains no free Cys residue, three single-Cys mutants were prepared: Q27C, E86C, S158C by use of the QuikChange Site-Directed Mutagenesis Kit (Stratagene, La Jolla, CA, USA) as previously described [18]. The mutated plasmids were confirmed by DNA sequencing and their recombinant proteins were subsequently expressed and purified by the same procedures described above. ^1^H-^15^N heteronuclear single quantum coherence spectroscopy (HSQC) experiments were performed on each mutant to validate that these mutations did not significantly perturb the conformation of the native NS3pro sequence.

The recombinant proteins of three single-cysteine mutants were Cys-modified following the previous procedure [18], by the thiol-reactive nitroxide free radical probe, MTSSL (1-oxyl-2,2,5,5-tetramethyl-Δ3-pyrroline-3-methyl) methanethiosulfonate (Toronto Research Chemicals Inc.). Briefly, the HPLC-purified recombinant protein of the each mutant was dissolved in the buffer containing 8 M urea, 20 mM phosphate (pH 8.0), which was pre-degassed with nitrogen gas for 20 minutes. Subsequently, the MTSSL reagent was added from 3.8 mM stock solution in acetonitrile to reach a six-fold molar concentration of the protein, followed by incubation at room temperature with constant stirring for 5 hours. To ensure a complete labeling, another dose of MTSSL was added to a six-fold molar concentration of the protein for an overnight incubation. The MTSSL-labeled protein was purified by reverse-phase HPLC on a C8 column and lyophilized. Based on the verification by the time-of-flight-mass spectrometer, the purity of the MTSSL-modified proteins of all mutants was > 99% after the HPLC purification.

### CD and NMR experiments

All circular dichroism (CD) experiments were performed on a Jasco J-810 spectropolarimeter equipped with a thermal controller using 1-mm path length cuvettes. Data from five independent scans were added and averaged [18]. The NS2B, NS3pro and NS2B-NS3pro samples were prepared at a protein concentration of 20 μM in either Milli-Q water (pH 4.0) and 1 mM phosphate (pH 7.5) respectively. Secondary structure contents of different samples were obtained by decovoluting CD spectra with CONTINLL program (http://lamar.colostate.edu/~sreeram/CDPro/main.html).

All NMR experiments were acquired on an 800 MHz Bruker Avance spectrometer equipped with pulse field gradient units as described previously [18]. For characterizing the conformation of the isolated NS3pro in water, a pair of triple-resonance experiments HNCACB, CBCA(CO)NH as well as ^15^N-edited HSQC-TOCSY and HSQC-NOESY were collected for the sequential assignment on a ^15^N-/^13^C-double labelled or ^15^N-labelled sample at a protein concentration of 500 μM in 90% H_2_O/10% D_2_O (pH 4.0). For the refolded NS2B-NS3pro complexes, HSQC spectra were collected for ^15^N-labeled samples in either acetate buffer (pH 4.0), or phosphate buffer (pH 7.5) in the absence and in the presence of inhibitor *p*-Nitrophenyl-*p*-guanidino benzoate [11].

For assessing the backbone dynamics on the ps-ns time scale, {^1^H}-^15^N steady-state NOEs were obtained by recording spectra on the ^15^N-labeled NS3pro domain at 500 μM in either Milli-Q water (pH 4.0), with and without ^1^H presaturation with duration of 3 s plus a relaxation delay of 6 s at 800 MHz. All NMR data were processed with NMRPipe [24] and analysed with NMRView [25]. NH, N, Cα and Cβ chemical shifts of the isolated NS3pro in water at pH 4.0 were further analyzed by both Delta2D [26] to derive the secondary structure population.

### Paramagnetic relaxation enhancement (PRE) experiments

For each spin-labeled single-cysteine mutant, a pair of 2D ^1^H-^15^N HSQC spectra were acquired at a protein concentration of 150 μM in Milli-Q water (pH 4.0): one for the spin-labeled sample in the paramagnetic form, and another after adding ascorbic acid (to 10 mM) to the sample to reduce the nitroxide, yielding the diamagnetic sample. We also acquired HSQC spectra for 3 corresponding cysteine mutants without spin-labelling at the same conditions and only several HSQC peaks slightly shifted after spin-labeling, indicating that the spin-labeling would not significantly change the conformation. The spectra were subsequently analyzed to obtain intensity ratios of HSQC peaks in the paramagnetic and diamagnetic forms using the programs nmrPipe [24]. Protein structures were displayed by PyMol molecular graphics system (W. L. DeLano, DeLano Scientific LLC, San Carlos, CA).

### Molecular dynamics (MD) simulations

The crystal structure of the NS2B-NS3pro complex (PDB code: 2FOM) with an open conformation [7] was selected for molecular dynamics simulations. As NS2B residues Thr77-Met84 are missing in the crystal structure, those residues were added and the obtained structure was post-processed as previously described [20,21]. The simulation cell is a periodic cubic box with a minimum distance of 10 Å between the protein and the box walls to ensure the protein would not directly interact with its own periodic images given the cutoff. The water molecules, described using the TIP3P model, were filled in the periodic cubic box for the all atom simulation. 6 Na^+^ ions were randomly placed to neutralize the charge in MD system.

Three independent 20-ns MD simulations for either NS2B-NS3pro complex or NS3pro alone were performed with the program GROMACS [27] with the AMBER-03 [28] all-atom force field. The long-range electrostatic interactions were treated using the fast particle-mesh Ewald summation method [29], with the real space cutoff of 9 Å and a cutoff of 14 Å was used for the calculation of van der Waals interactions. The temperature during the simulations was kept constant at 300 K by Berendsen's coupling. The pressure was held at 1 bar. The isothermal compressibility was 4.5*10^−5^ bar^-1^. The time step was set as 2 fs. All bond lengths including hydrogen atoms were constrained by the LINCS algorithm [29]. Prior to MD simulations, all the initial structures were relaxed by 500 steps of energy minimization using the steepest descent algorithm, followed by 100 ps equilibration with a harmonic restraint potential applied to all the heavy atoms of the protease.

### Correlation analysis

As the 20-ns simulations cannot reproduce the folding-unfolding event, so here we attempted to capture the low-frequency correlation motions by a recently established approach called MutInf [30]. MutInf represents an entropy-based approach to analyze ensembles of protein conformers, such as those from molecular dynamics simulations by using internal coordinates and focusing on dihedral angles. In particular, this approach is even applicable for detecting conformational changes which are subtle in the short MD simulations because the coupling is mostly entropic in nature [30]. Briefly, this approach utilizes second-order terms from the configurational entropy expansion, called the mutual information, to identify pairs of residues with correlated conformations, or correlated motions, in an equilibrium ensemble [30]. In the present study, the normalized matrix values were used, and 0.3 was set up to be the threshold value to determine the pairs of highly correlated residues. The residue pairs with correlated values ≥ 0.3 make up the top 1% of the entire matrix.

## Results

### Solution conformations of the isolated NS2B and NS3pro

DNA fragments encoding NS2B (48–100) and NS3pro (14–185) were sub-cloned into the expression vectors pGEX-4T1 and pET-28 respectively and subsequently expressed in *E*. *coli* BL21 (DE3) Star cells. The recombinant NS3pro protein was found to be completely insoluble and all in inclusion body as previously reported [7,11]. As a consequence, the NS3pro proteins were first purified by Ni^2+^-affinity column under denaturing condition in the presence of 8 M urea, followed by purification with reverse-phase (RP) HPLC. On the other hand, the GST-fused NS2B (48–100) cofactor was found in supernatant and thereby purified under native condition, followed by the thrombin cleavage to release the cofactor which was also purified by RP-HPLC.

**Fig 1:**
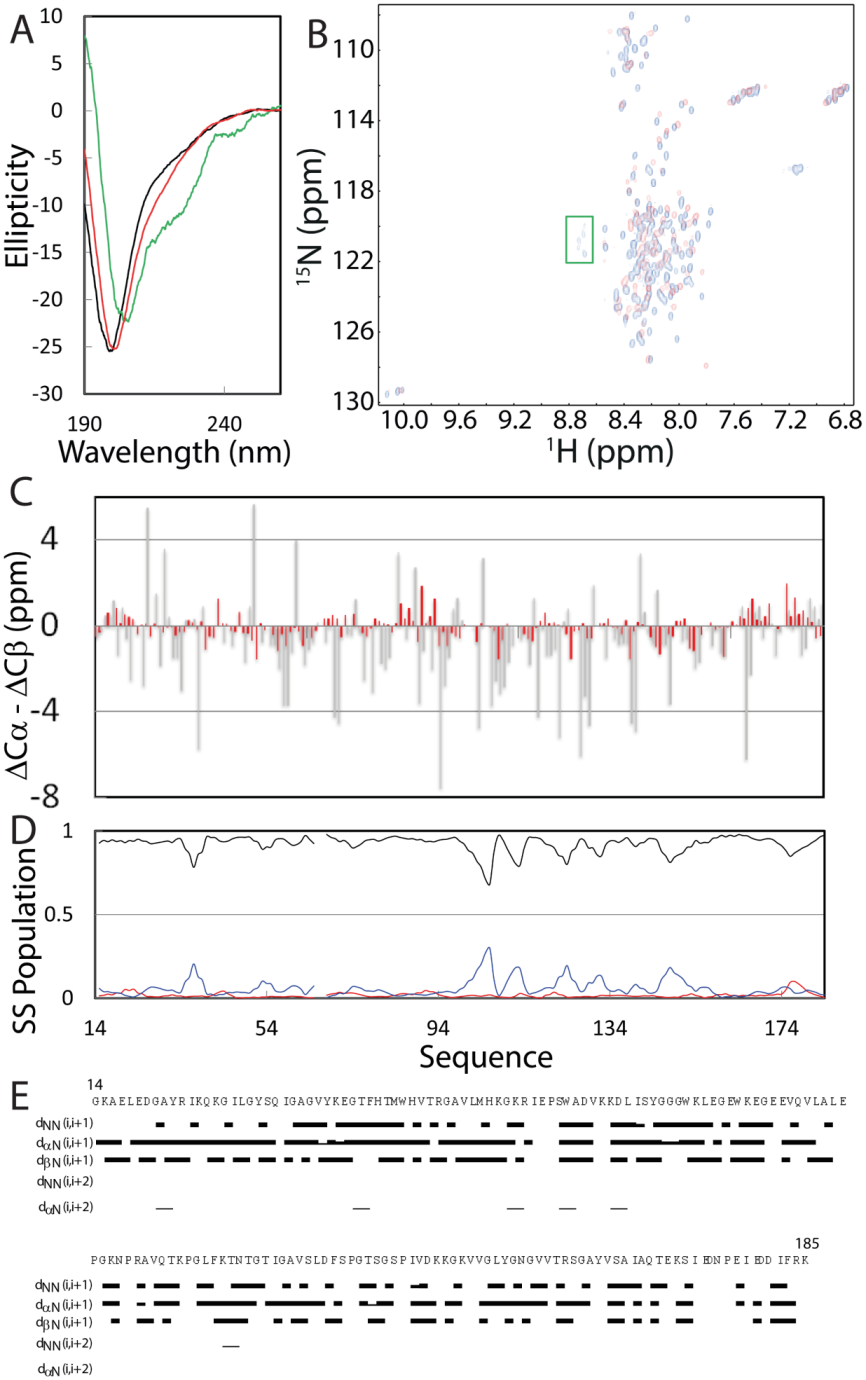
Characterization of the solution conformations of the isolated NS2B and NS3pro. (A) Far-UV CD spectra of the isolated NS2B (green), NS3pro at a protein concentration of 20 μM in Milli-Q water at pH 4.0 (black) and in 5 mM phosphate buffer at pH 7.0 (red). (B) Two-dimensional NMR ^1^H-^15^N HSQC spectra of the isolated NS3pro at a protein concentration of 100 μM in Milli-Q water at pH 4.0 (blue) and in 5 mM phosphate buffer at pH 7.0 (red). (C) Residue specific (ΔCα-ΔCβ) values of NS3pro in the isolated state obtained in the present study (red) and in the complex with NS2B (grey) previously published (10). (D) Secondary structure populations obtained by analyzing chemical shifts of the isolated NS3pro domain with the Delta2D program. Red line is used for indicating the population of helix; blue for extended strand and black for random coil. (E) NOE connectivities defining secondary structures of the isolated NS3pro.

The lyophilized powder of the NS2B (48–100) was soluble both in Mill-Q water and in buffers, while the NS3pro protein was highly soluble at protein concentration of 1 mM without any detectable aggregation for at least half a year in Mill-Q water. The NS3pro protein also could be quickly diluted into 5 mM phosphate buffer with a final protein concentration of 100 μM and pH of 7.0 without visible aggregates for 1 hr, but formed aggregates in NMR tube after 4 hr, which has been commonly observed on a variety of “completely insoluble” proteins previously reported by us and other groups [15–18]. As shown in Fig 1A, NS3pro has very similar far-UV CD spectra in Milli-Q water (pH 4.0) and 5 mM phosphate buffer (pH 7.0), with the maximal negative signal at 200 nm and no positive signal below 200 nm, which is typical of a predominantly disordered protein without any stable secondary structure. This indicates that the isolated NS3pro domain is highly disordered in aqueous solution. Indeed, the deconvolution of its CD spectrum reveals that the isolated NS3pro consists of 53% random coil, 14% turn, 29% extended strand and 4% helix secondary structures. Moreover, it has a HSQC spectrum with very narrow spectral dispersions at both ^1^H (~0.9 ppm) and ^15^N (~19 ppm) dimensions (Fig 1B), further indicating the absence of tight tertiary packing.

Interestingly, on the other hand, NS2B (48–100) has a CD spectrum with the maximal negative signal at 206 nm and positive signal at 190 nm, as well as an additional negative signal at 222 nm. Further deconvolution shows that it contains 32% random coil, 24% turn, 7% extended strand and 37% helix secondary structures. Previously NS2B has been shown to have slightly different secondary structures in the open [7] and closed [8] states of the NS2B-NS3pro complexes. In the open (inactive) state (PDB ID: 2FOM), NS2B has a short helix over residues Glu62-Gly69 while in the closed (active) state (PDB ID: 3U1I), the NS2B only has β-strands but no helical segment. Therefore, it is possible that upon losing tertiary contacts with the NS3pro domain, the free-state NS2B has more helical conformations populated over residues Glu62-Gly69, or/and even has helical conformations populated over residues which adopt β-strands in the NS2B-NS3pro complexes. Indeed, we previously observed that upon disrupting the all β-barrel native fold, the mutants of a SH3 domain became highly helical even over the residues which adopt β-strands in the native fold [31]. Unfortunately, HSQC peaks of the NS2B (48–100) are much more broadened than those of NS3pro, implying that the isolated NS2B (48–100) has dynamic aggregation or/and conformational exchanges on the μs-ms time scale, thus preventing from further high-resolution NMR studies.

### Residue-specific conformation of the isolated NS3pro

Despite its narrow spectral dispersions, we have successfully assigned NMR resonances of almost all non-proline residues of the 172-residue NS3pro (14–185) by analyzing NMR spectra including HN(CO)CACB/CBCA(CO)HN and HSQC-TOCSY/HSQC-NOESY. Fig 1C presents the obtained (ΔCα-ΔCβ) chemical shifts, which is a sensitive indicator of the residual secondary structures in disordered proteins [32]. Previously, by analyzing NMR chemical shifts, the NS2B (48–100)-NS3pro (14–185) complex was characterized to adopt the same structure in solution and crystal [10]. Here we downloaded the chemical shifts of NS3pro in complex with NS2B (BMRB ID of 19080) and included them (grey bars in Fig 1C) in parallel to those of the isolated NS3pro (red bars in Fig 1C) for comparison.

The NS3pro domain in complex with NS2B has very large (ΔCα-ΔCβ) deviations characteristic of a well-folded protein. By contrast, the isolated NS3pro has dramatically decreased (ΔCα-ΔCβ) over the whole sequence (Fig 1C). For example, many NS3pro residues in the complex have the absolute values of (ΔCα-ΔCβ) > 4 ppm, while all isolated NS3pro residues have the absolute values of (ΔCα-ΔCβ) < 2 ppm. Interestingly, many residues of the isolated NS3pro still have the (ΔCα-ΔCβ) patterns similar to those in the complex, implying that similar secondary structures might be weakly populated over these residues. Indeed, we further analysed the NH, ^15^N, Cα and Cβ chemical shifts by Delta2D program [26] and the results indicate that the isolated NS3pro adopts highly-populated random coil conformations over the whole sequence. Nevertheless, over some short segments, the extended strand conformation is also populated to some degree but the helix conformation is almost lacking (Fig 1D), completely consistent with the deconvolution result of the CD spectrum. Furthermore, as seen in Fig 1E, only sequential NOEs d_NN(i,i+1)_ and d_αN(i,i+1)_ manifest over the majority of the sequence, clearly indicating that the isolated NS3pro has no stable secondary structures and its NMR conformation represents an average of an ensemble of different structures. Taken together, CD and NMR results define the 172-residue NS3pro domain to be an intrinsically disordered protein which is lacking of both stable secondary and tertiary structures in the absence of the NS2B cofactor [22,31–36].

### Backbone dynamics and long-range interactions of the isolated NS3pro

**Fig 2:**
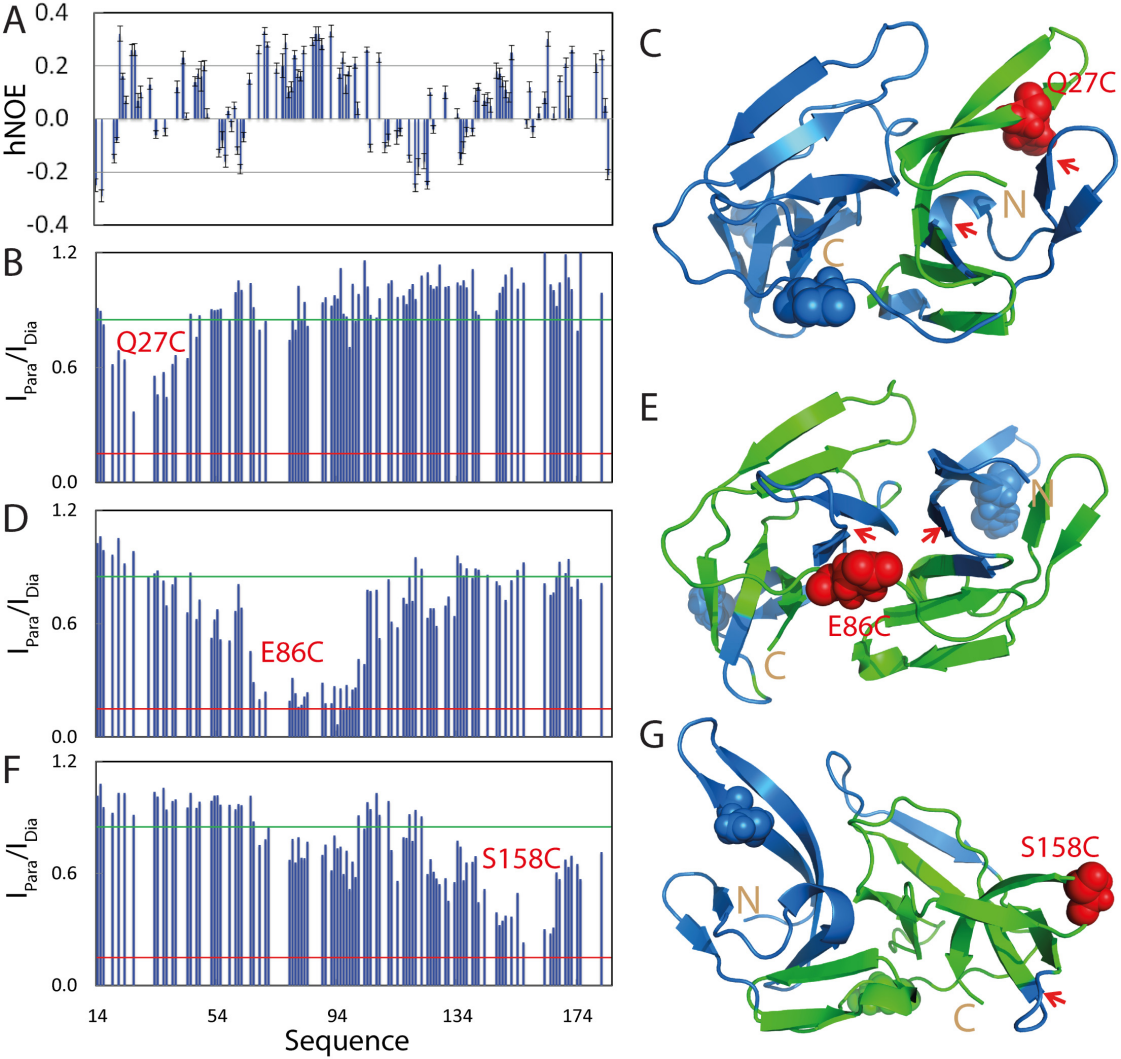
Backbone dynamics and long-range interactions in the isolated NS3pro. (A) {^1^H}–^15^N heteronuclear steady-state NOE (hNOE) of the isolated NS3pro. Intensity ratios of HSQC peaks of spin-labeled Q27C (B), E86C (D) and S158C (F) in the paramagnetic state of the MTSL probe and diamagnetic state after the MTSL probe was reduced. NS3pro structure with the residue colored in green if its intensity ratio <0.85 for Q27C (C), E86C (E) and S158C (G).

To pinpoint the backbone flexibility, we measured the {^1^H}-^15^N heteronuclear steady-state NOE (hNOE) of the isolated NS3pro, which is a measure of the backbone motions on the ps-ns time scale [18,19,31,32,37]. Previously, all except for several C-terminal residues of the complexed NS3pro were shown to have positive hNOE values, with many even close to 1, clearly indicating that the backbone of the NS3pro in the complex is very rigid [10]. By contrast, residues of the isolated NS3pro have small or even negative hNOEs (Fig 2A), with an average hNOE of only 0.08. More specifically, all residues have hNOE values < 0.4, while many residues even have negative hNOE, such as N-/C-termini, Ser34-Gly39, Arg54-Arg64, Gln110-Ile139 and Arg157-Gly159 (Fig 2A). This strongly suggests that the isolated NS3pro has largely unrestricted backbone motions on the ps-ns time scale, consistent with its absence of any stable secondary and tertiary structures. Nevertheless, residues over Ile65-Leu100 have relatively large hNOE, implying that this region might have transit tertiary packing to a certain degree.

Previously many intrinsically disordered proteins such as α-synuclein have been shown to have transient long-range interactions by measurements of paramagnetic relaxation enhancement (PRE), which is a powerful tool in detecting transiently existing contacts in highly disordered proteins with distances up to ~25 Å [18,32,38]. Here, by site-directed mutagenesis, we introduced Cys residue into NS3pro one by one at three locations: Gln27, Glu86 and Ser158 (S1 Fig). Three single Cys mutants were subsequently labelled with the nitroxide free radical probe, MTSSL whose PRE were measured by HSQC (S1 Fig) as we previously described [18]. Fig 2B shows the intensity ratios of HSQC peaks from the oxidized (paramagnetic) and reduced (diamagnetic) spectra of Q27C mutant. Interestingly, the residues which were significant affected (ratio < 0.85) are all located in the first β-barrel of the chymotrypsin fold adopted by the NS3pro domain in the native NS2B-NS3pro complex (Fig 2C). This suggests that these affected residues have long-range contacts with Cys27 with distances < 25 Å. Interestingly, although residues Thr48-Glu66 assuming a short helix and β-sheet have very close contacts with Cys27 in the native structure, they were not significantly perturbed by the spin-label at Cys27. This strongly implies that the tertiary packing is non-native in the isolated NS3pro domain. The spin-label at the position 86 significantly affected the residues on both β-barrels (Fig 2D and 2E), suggesting that these residues have long-range contacts to Cys86 with distances < 25 Å. Again, many residues have very close contacts with Cys86 in the native fold but were not significantly perturbed by the spin-label at Cys88. The residues affected by the spin-label at the position 158 are mostly located on second β-barrel, the loop connecting two β-barrels, and the last β-sheet over residues Ser68-Gly71 (Fig 2F and 2G).

The PRE measurements revealed that despite lacking of stable secondary and tight tertiary structures, the isolated NS3pro domain is not completely extended, but instead has a loose tertiary packing. However, this packing is highly dynamic and non-native, in which the residues over the middle region of the NS3pro sequence are slightly less dynamic than the N- and C-terminal ones, completely consistent with hNOE results (Fig 2A).

### Structure and activity of the refolded NS2B-NS3pro complexes

**Fig 3:**
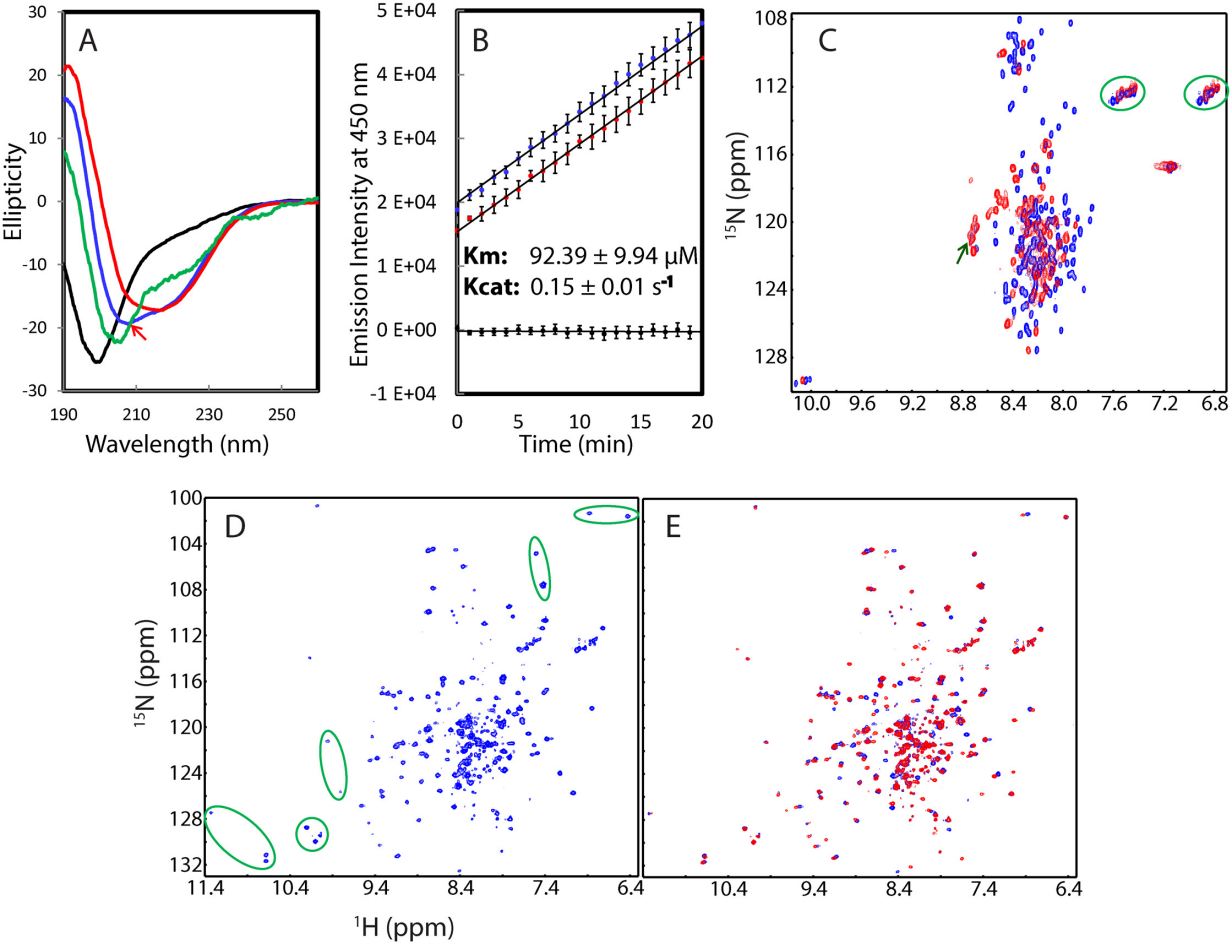
Conformations and activity of the refolded NS3pro in complex with NS2B(40–100). (A) Far-UV CD spectra of the isolated NS3pro (black), NS2B (48–100) (green), refolded NS2B (48–100)-NS3pro complex at a molar ratio of 1:1 at Milli-Q water at pH 4.0 (blue) and in the buffer at pH 7.5 (red). (B) Enzymatic activities of the isolated NS3pro (black), NS2B-NS3pro complex refolded at pH 4.0 (blue) and at 7.5 (red) as measured in the assay buffer (pH 7.5) by monitoring the increase of the emission fluorescence intensity at a wavelength of 450 nm. The Km and kcat values are presented for the complex refolded at pH 7.5. (C) HSQC spectra of the isolated NS3pro (blue) and NS2B-NS3pro mixture (red) in Milli-Q water (pH 4.0). (D) HSQC spectrum of the refolded NS2B-NS3pro complex in which only the NS3pro is ^15^N-labeled in buffer (pH 7.5). Green ovals are used to indicate characteristic peaks also observed in the previous reports (refs. 10 and 11). (E) HSQC spectra of the NS2B-NS3pro in buffer (pH 7.5) in the absence (blue) and in the presence of the protease inhibitor *p*-Nitrophenyl-*p*-guanidino benzoate (red).

The isolated NS3pro (14–185) and NS2B (48–100) are largely disordered in solution (Fig 3A) and the NS3pro alone showed no detectable catalytic activity even with a protease concentration up to 5 μM in buffers at pH 7.5 (Fig 3B). However, upon mixing them at an equal molar ratio in Milli-Q water at pH 4.0, the mixture has a CD spectrum for a protein with a substantial amount of secondary structures, which has a large positive signal at 190 nm and the maximal negative signal at 208 nm (Fig 3A). The deconvolution shows that this refolded complex between NS2B (48–100) and NS3pro (14–185) in water at pH 4.0 contains a large portion of β-strand and β-turn structures: 34% random coil, 22% turn, 37% extended strand and 7% helix secondary structures, which is consistent with its three-dimensional structure (Fig 2).

This sample was subsequently split into two: one at the same condition at pH 4.0, and another with buffer added to reach pH 7.5. As shown in Fig 3A, the CD spectrum of the mixture in buffer at pH 7.5 has slight changes: the maximal positive signal shifted to 192 nm as well as became larger; and the maximal negative signal shifted from 208 to 217 nm, almost identical to that previously reported on the co-expressed NS2B-NS3pro complex [10]. This change appears to result from the slight increase of β-strand and reduction of random coil conformations.

The slight variations in CD spectra of the refolded NS2B-NS3pro complex at pH 4.0 and 7.5 suggest that the complex may have some conformational differences at two pH values. Indeed, the complex at pH 4.0 has a HSQC spectrum with many HSQC peaks too broad to be detected (Fig 3C), indicating that the complex undergoes significant dynamic aggregation, or/and conformational exchanges on the μs-ms time scale. By contrast, the refolded NS2B-NS3pro complex at pH 7.5 has a HSQC spectrum typical of a well-folded protein, which has very large spectral dispersions at both ^1^H (~3.6 ppm) and ^15^N (~28 ppm) dimensions (Fig 3D). In particular, although in the present study the NS2B peptide is unlabelled, a large portion of the HSQC peaks of the present NS3pro domain are almost superimposable to those of the previous complex with both NS2B and NS3pro ^15^N-labelled and co-expressed [10,11], as exemplified by the characteristic HSQC peaks (green cycled), Furthermore, the addition of the protease inhibitor *p*-Nitrophenyl-*p*-guanidino benzoate previously used [11] triggered dramatic shifts of many HSQC peaks, indicating the refolded NS2B-NS3pro is active in binding the inhibitor (Fig 3E).

We measured the enzymatic activity of the complex refolded at pH 4.0 in two buffers: one at pH 4.0 and another at pH 7.5. Interestingly, it has no detectable activity in the acetate buffer at pH 4.0 even with the protein concentration reaching 5 μM. Nevertheless, if the sample refolded at pH 4.0 was measured in the buffer at pH 7.5, it is highly active with no detectable difference from that refolded in the buffer at pH 7.5 (Fig 3B). The enzymatic parameters of the refolded NS2B-NS3pro complex in the buffer at pH 7.5 were obtained with Km = 92.39 ± 9.94 μM and Kcat = 0.15 ± 0.01 s^-1^, which are very similar to the previous results with a co-expressed NS2B-NS3pro complex [10].

The structure and enzymatic activity revealed that once NS2B and NS3pro meet, they will bind to each other and initiate the folding process to form the two-component complex. However, it seems that if at pH 4.0, the NS2B-NS3pro complex exists as an intermediate which already has substantial secondary structures and tertiary packing. However, the tight tertiary packing is not completely achieved and consequently the complex undergoes μs-ms conformational exchanges, very similar to what we previously observed on a molten globule formed by a small protein at pH 4.0 [39,40]. The intermediate of the NS2B-NS3pro complex at pH 4.0 is enzymatically inactive, but nevertheless, once it is transferred into the buffer at pH 7.5, the tightly-packed structure will immediately form and the complex can reach the fully active state.

### Structure and activity of the NS3pro in complex with the full-length NS2B (1–130) anchored in the LMPC micelle

To study the structure and activity of the NS3pro in complex with the full-length NS2B (1–130), which is expected to be anchored into the membranes by forming the transmembrane helices at both N- and C-termini, the DNA fragment encoding the full-length NS2B (1–130) was cloned into pET28a vector and the NS2B (1–130) protein was purified under denaturing condition. The full-length NS2B (1–130) was reconstituted in the LMPC micelle at a molar ratio of 1:200 (NS2B:LMPC) in buffer (pH 7.5). Furthermore, NS3pro and NS2B (1–130) were also refolded together at the equimolar concentrations in presence of LMPC in buffer (pH 7.5).

**Fig 4:**
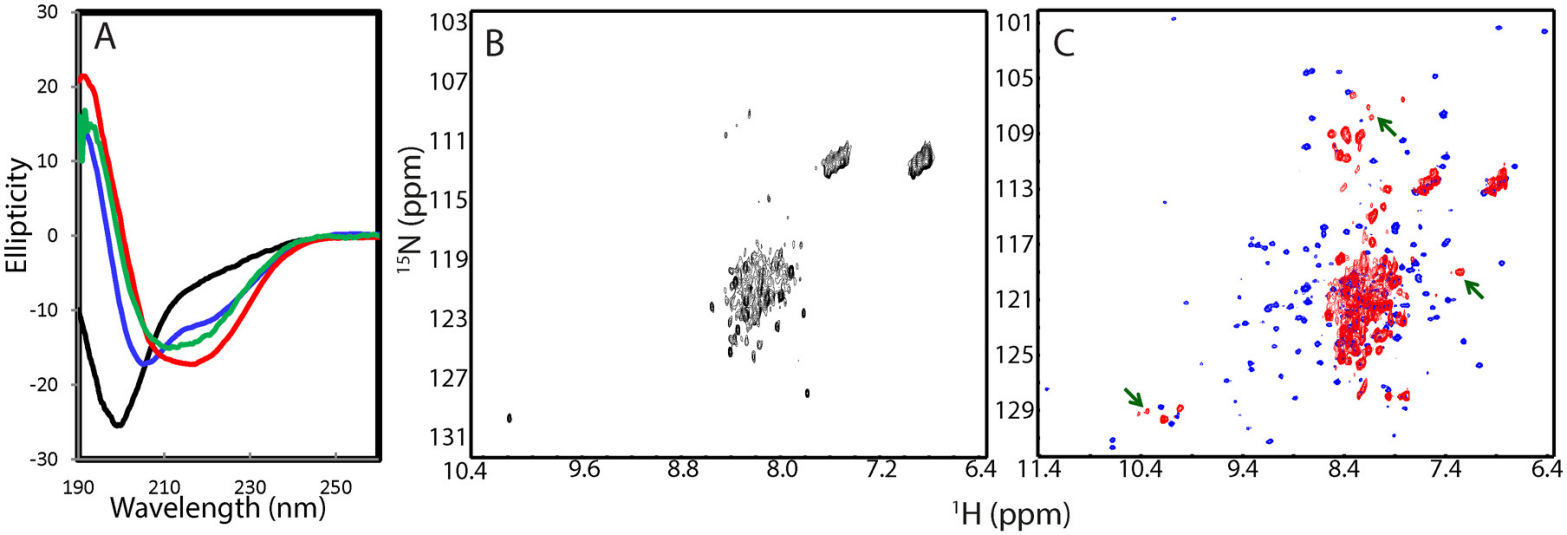
Conformations of the refolded NS3pro in complex with NS2B (1–130) in the LMPC micelle. (A) Far-UV CD spectra of the isolated NS3pro (black), NS2B (1–130) reconstituted in the LMPC micelle, refolded NS2B (48–100)-NS3pro complex at a molar ratio of 1:1 in the buffer at pH 7.5 (red), and refolded NS2B (1–130)-NS3pro complex reconstituted in the LMPC micelle in the buffer at pH 7.5 (blue). (B) HSQC spectrum of the full-length NS2B (1–130) reconstituted in the LMPC micelle in the buffer at pH 7.5. (C) HSQC spectra of the NS2B (48–130)-NS3pro complex in buffer (pH 7.5) (blue) and refolded NS2B (1–130)-NS3pro complex reconstituted in the LMPC micelle in the buffer at pH 7.5 (red). The green arrows are used to indicate the HSQC peaks of the refolded NS2B (1–130)-NS3pro complex which are not superimposable with those of the NS2B (48–130)-NS3pro complex.

Interestingly, the far-UV CD spectrum of the full-length NS2B (1–130) reconstituted in the LMPC micelle have the maximal negative signal at 209 nm and positive signal at 192 nm, as well as an additional negative signal at 222 nm (Fig 4A). The deconvolution reveals that the NS2B (1–130) reconstituted in the LMPC micelle contains 28% random coil, 23% turn, 17% extended strand and 32% helix secondary structures. The large portion of the helix conformation is anticipated to result from the formation of the transmembrane helices at both of N- and C-terminal termini. Interestingly, the NS2B (1–130) in the LMPC micelle has ~10% higher β-strand conformation than the NS2B (48–100) in buffer, implying that they only have a slight difference in secondary structures if considering the error in CD deconvolution. On the other hand, only a small set of broad peaks could be detected in its HSQC spectrum (Fig 4B), indicating that the NS3B (1–130) in the LMPC micelle undergoes significant conformational exchanges on μs-ms time scale, or/and dynamic aggregation, which thus prevents from further high-resolution NMR studies. The observed line-broadening cannot mainly result from the increased molecular weight in the LMPC micelle as much more narrow peaks for almost all residues could be detected for the helical superoxide dismutase 1 (SOD1) with 153 residues in the DPC micelle [41].

We also refolded the NS3pro with the full-length NS2B (1–130) in the LMPC micelle. As shown in Fig 4A, the far-UV CD spectrum of the complex between NS2B (1–130) and NS3pro in the LMPC micelle has the maximal negative signal at 206 nm and positive signal at 190 nm, as well as an additional negative signal at 222 nm (Fig 4A). The deconvolution shows that its secondary structure contents are very similar to those of the complex between NS2B (48–100) and NS3pro in buffer, only with ~3% increase of β-strand conformation and ~1.6% reduction of the helix conformation. However, very different from the complex between NS2B (48–100) and NS3pro in buffer (Fig 3D), for the HSQC spectrum of the complex between NS2B (1–130) and NS3pro in the LMPC micelle (Fig 4C), only a small portion of the HSQC resonance peaks could be observed, which are also much more broad. This is partly due to the increased molecular weight of the complex upon being anchored into the LMPC micelle. A closer examination of the spectra revealed that a small set of the NS3pro HSQC peaks of the NS2B (1–130)-NS3pro complex in the LMPC micelle is almost superimposable to those of the NS2B (40–100)-NS3pro complex. These residues were identified to be the C-terminal residues Glu173-Lys185 of NS3pro, which were not visible in crystal structures of the NS2B-NS3pro complexes, thus suggesting that they are similarly flexible in both complexes NS2B (40–100)-NS3pro in buffer and NS2B (1–130)-NS3pro in the LMPC micelle). On the other hand, some HSQC peaks of the NS3pro domain complexed with the NS2B (1–130) in the LMPC micelle are not superimposable to those in the NS2B (40–100)-NS3pro complex. This implies that these NS3pro residues may have different structures, or/and dynamics, or/and chemical environments in the NS2B (40–100)-NS3pro and NS2B (1–130)-NS3pro in the LMPC micelle. Furthermore the fact that most well-dispersed HSQC peaks disappeared (Fig 4C) implies that the NS3pro may undergoes significant conformational exchanges on μs-ms time scale upon being anchored in the LMPC micelle. Nevertheless, the NS2B (1–130)-NS3pro in the LMPC micelle is similarly active, with Km = 101.1 ± 3.163 μM and Kcat = 0.2469 ± 0.003 s^-1^, which are very similar to the activity of the NS3pro in complex with the NS2B (48–100) with transmembrane regions deleted.

### Molecular dynamics (MD) simulations

Molecular dynamics simulation is a powerful tool to gain insights into protein dynamics and folding/unfolding that underlies protein functions. Although it remains extremely challenging to simulate the folding/unfolding for large proteins such as NS3pro, which usually occurs on the ms-s time scale, short MD simulations in conjunction with the correlation analysis are able to capture the low-frequency correlated motions, which have been recently found to be essential for the catalysis of the SARS 3C-like protease [20,21]. Therefore, to understand the role of the cofactor in the dynamics of the dengue NS3 protease, we conducted 20-ns MD simulations for both NS2B-NS3pro complex and isolated NS3pro respectively. Here we used the crystal structure of the NS2B-NS3pro complex (PDB code: 2FOM) with an open conformation [7] for MD simulations, because this complex has sequences almost identical to what we studied here, with only one and two conserved residue variations respectively as compared to our NS3pro and NS2B constructs. Furthermore, in this structure, the NS2B cofactor assumes an open conformation, thus has a minimal contact surface with the NS3pro domain as compared to other structures with the closed conformation. This would facilitate the identification of the most important residues of the NS2B in mediating the dynamics of the protease complex.

**Fig 5:**
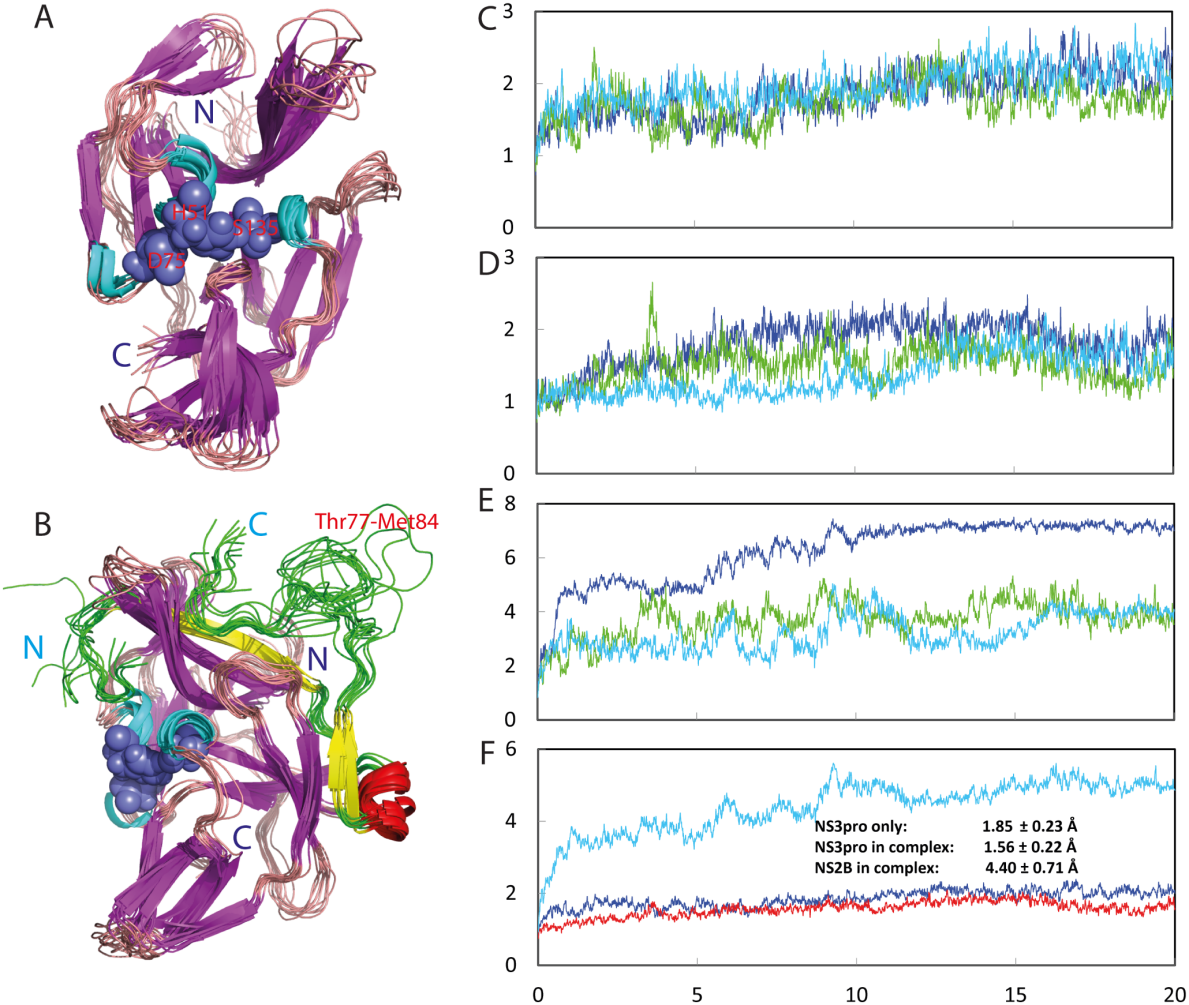
Overall dynamic behaviors in the MD simulations. Structure snapshots (one structure for 2-ns interval) of the first MD simulations respectively for the isolated NS3pro (A) and NS2B-NS3pro complex (B). Root-mean-square deviations (RMSD) of the Cα atoms (from their positions in the energy minimized structures) for three independent MD simulations of the isolated NS3pro (C), the NS3pro (D) and NS2B (E) in the context of the NS2B-NS3pro complex. (F) RMSD trajectories averaged over three independent MD simulations of the isolated NS3pro (blue), the NS3pro (red) and NS2B (cyan) in the context of the NS2B-NS3pro complex.

Fig 5A and 5B present the structure snapshots in the first MD simulations for the NS3pro alone and the NS2B-NS3pro complex, showing that within 20 ns, both isolated NS3pro domain and that in the complex remains dynamically stable, in particular over the regions with regular secondary structures. By contrast, the NS2B cofactor has large structural fluctuations, particularly over the residues Thr77-Met84 which are invisible in all previous crystal structures with an open conformation. Fig 5C–5E show the root-mean-square deviations (RMSD) of Cα atoms (from their positions in the energy minimized structures) for three independent simulations of the isolated NS3pro, the NS2B and NS3pro in the context of the complex respectively, and Fig 5F shows their averaged RMSD trajectories. The averaged RMSD values are 1.85 ± 0.23, 1.56 ± 0.22 and 4.40 ± 0.71 Å respectively for the isolated NS3pro, the NS3pro and NS2B in the context of the complex. This clearly indicates that upon removing NS2B, the isolated NS3pro domain will have higher conformational dynamics even within the 20-ns simulations. As such, it is expected that the isolated NS3pro domain may become unfolded if the simulations could reach ms-s time scale. On the other hand, even in the context of the complex, the NS2B cofactor has much higher structural fluctuations than NS3pro, implying that the bound NS2B cofactor is still capable to sample a large ensemble of conformations. The high flexibility of NS2B uncovered by MD simulations here is completely consistent with the invisibility of many cofactor residues in crystal structures and also provides a dynamic basis for the conformational exchange of NS2B between open and closed conformations as previously observed [8–12].

**Fig 6:**
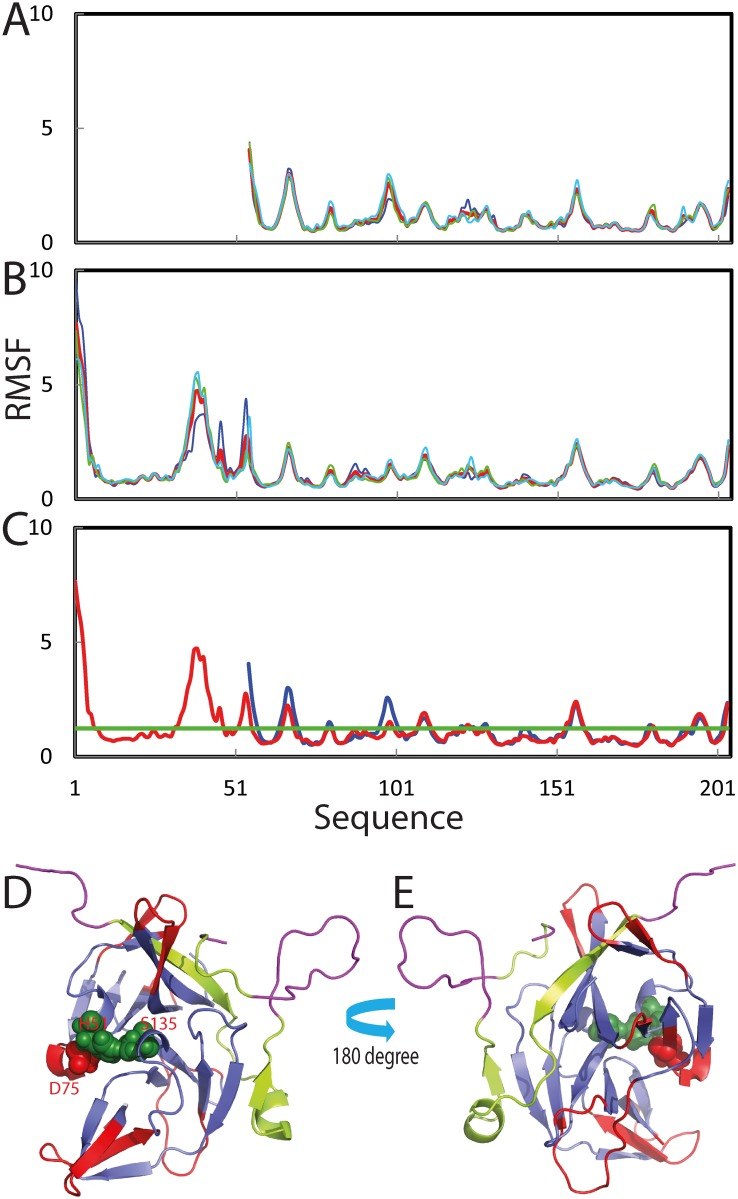
Residue-specific dynamic behaviors in the MD simulations. Root-mean-square fluctuations (RMSF) of the Cα atoms for three independent MD simulations of the isolated NS3pro (A) and the NS2B-NS3pro complex (B). (C) RMSF trajectories averaged over three independent MD simulations of the isolated NS3pro (blue) and the NS2B-NS3pro complex (red). (D)-(E) The NS2B-NS3pro complex structure with the residues colored as: red for the residues having RMSF > average value if in NS3pro, or purple if in NS2B.

Noticeably, similar dynamic behaviours are also reflected by the root-mean-square fluctuations (RMSF) of the Cα atoms in the MD simulations (Fig 6A and 6B). Fig 6C presents the averaged RMSF of three trajectories for the isolated NS3pro and NS2B-NS3pro complex. Interestingly, only three regions of the isolated NS3pro have slightly higher fluctuations than the corresponding ones in the complex. The highest structural fluctuations are observed on the NS2B residues. More specifically, the NS2B residues with RMSF > the average are over N-/C-terminal residues G43-Ala49 and Leu95-Gly96, as well as Ser75-Glu89 which include the missing residues Thr77-Met84 in all crystal structures of the open form [7]. Strikingly, the NS3pro residues with RMSF > the average are not only over loop/turn residues and C-terminal residues which include G29-Leu31, Glu43-Thr45, His60-Gly62, Leu85-Val95, Thr118-Gly121, Gly144-Val146 and Arg157-Gly159; but also over the short helix Val72-Lys74, and β-strands Gly32-Gln35, Lys63-Ile65, Asp75-Ile77 hosting the catalytic triad residue Asp75, Thr122-Val126 and Gly159-Val162 (Fig 6D and 6E).

**Fig 7:**
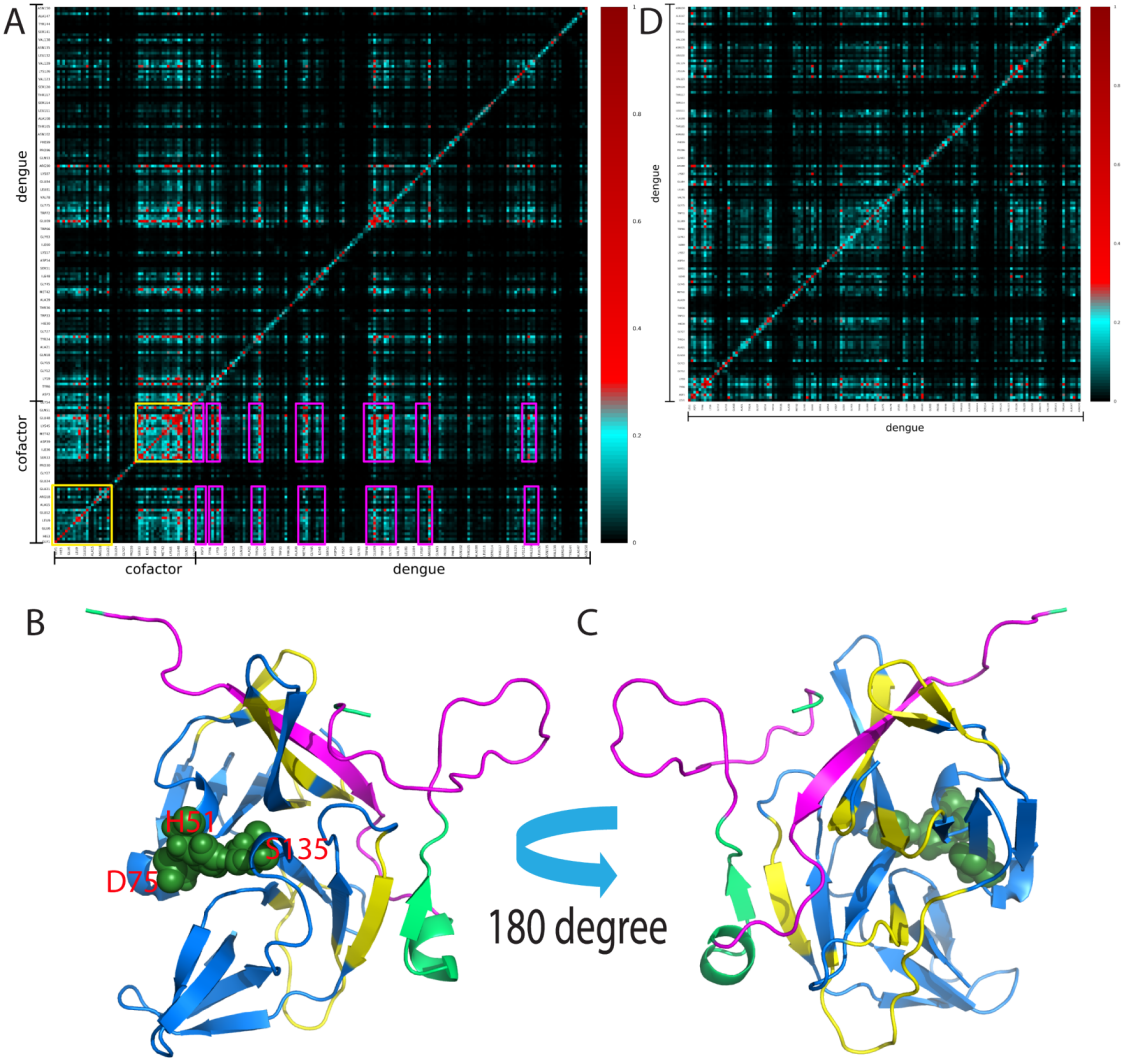
The existence of a global networks of correlated motions. Mutual information matrixes of the NS2B-NS3pro complex (A) and isolated NS3pro (B) calculated from their MD simulation data by MutInf. Yellow boxes are used for indicating the NS2B cofactor residues while pink boxes for the NS3pro residues having significant correlation motions. (C)-(D) The NS2B-NS3pro complex structure with the residues colored as: yellow for the residues having significant correlated motions if in NS3pro, or pink if in NS2B.

Recently, we have deciphered that a global correlation motion network exists in the SARS 3C-like protease [20,21]. Remarkably, a mutation N214A on the extra domain which is far away from the active site is sufficient to decouple the correlated motions and consequently leads to the inactivation of the enzymatic catalysis. Here, the correlation analysis of the MD simulation trajectories revealed that like the SARS 3C-like protease, a global correlation network does exist in the NS2B-NS3pro complex. Most strikingly, in this network, the majority of the significant correlated motions are established between the cofactor and NS3pro residues (Fig 7A). Surprisingly, although the cofactor residues Ser44-Glu63, Ser75-Thr94 are highly dynamic in the MD simulations, and Thr77-Met84 are even missing in all crystal structures of the open form, they were revealed to play a key role in coordinating the correlated motions with the NS3pro residues Asp20-Gln28, Gly39-Gly44, Met59-Glu66, Ly84-Val95, Pro106-Gln110, and Lys145-Gly148, which cover not only the residues having direct contacts with the cofactor, but also residues located far away from the cofactor (Fig 7B and 7C). Furthermore, the global correlation network is largely eliminated as uncovered by the correlation analysis of the MD trajectories of the isolated NS3pro with the NS2B cofactor removed (Fig 7D), which is similar to what was observed on the inactivated N214A mutant of the SARS 3C-like protease [19,20]. Therefore, as implied by our previous results with the SARS 3C-like protease, slight manipulations of the NS2B-NS3pro interface may be sufficient to decouple the correlation network to inactivate the activity of the dengue protease.

### The conformation and activity of the refolded NS3pro in complex with NS2B (48–100;Δ77–84)

**Fig 8:**
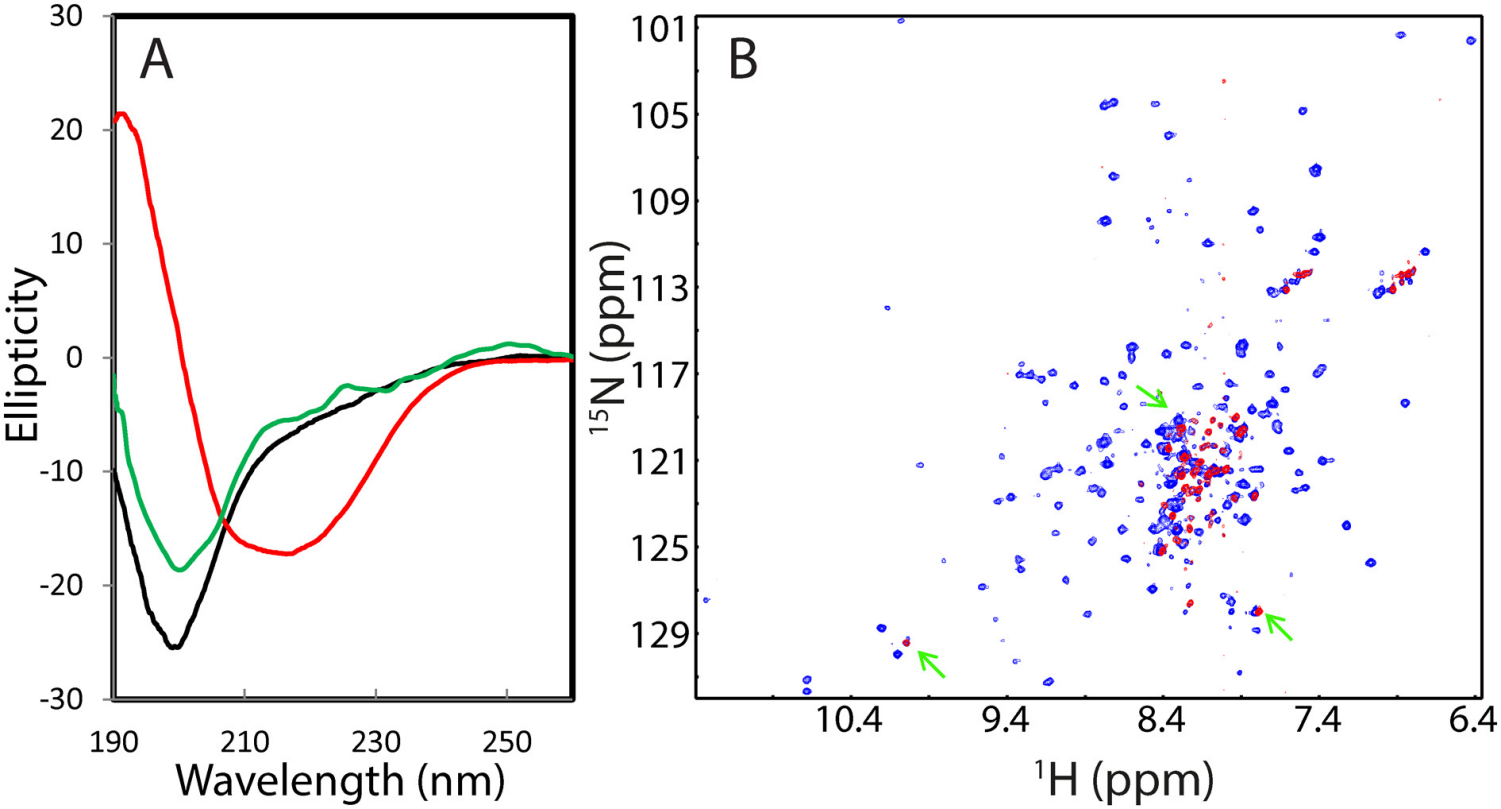
Conformation of the refolded NS3pro in complex with NS2B (48–100;Δ77–84). (A) Far-UV CD spectra of the isolated NS3pro (black), refolded NS2B (48–100)-NS3pro complex at a molar ratio of 1:1 in the buffer at pH 7.5 (red) and NS2B (48–100;Δ77–84)-NS3pro complex at a molar ratio of 1:1 in the buffer at pH 7.5 (green). (B) HSQC spectra of the refolded NS2B (48–100)-NS3pro complex in the buffer at pH 7.5 (blue) and NS2B (48–100;Δ77–84)-NS3pro complex in the buffer at pH 7.5 (red). Green arrows are used to indicate HSQC peaks of the NS2B (48–100;Δ77–84)-NS3pro complex which are superimposable to those of the NS2B (48–100)-NS3pro complex.

Although residues Thr77-Met84 are missing in all crystal structures of the open form, they were revealed by MD simulations to play a key role in coordinating the correlated motions with the NS3pro residues. Therefore, we generated a truncated NS2B (48–100;Δ77–84) in which the residues Thr77-Met84 were replaced by three Gly residues and subsequently conducted the refolding of NS2B (48–100;Δ77–84) with NS3pro using the same protocol for refolding of NS2B (48–100). Interestingly, although the isolated NS3pro is completely insoluble in buffer, the NS3pro became highly soluble in buffer at pH 7.5 in the presence of NS2B (48–100;Δ77–84). This suggests that NS3pro forms a complex with NS2B (48–100;Δ77–84) and the complex is soluble in buffer. Indeed, as shown in, the decovolution analysis of the far-UV CD spectrum (Fig 8A) reveals that the NS2B (48–100;Δ77–84)-NS3pro complex contains 19% helix, much higher than those of the isolated NS3pro (4%), and NS2B (48–100)-NS3pro complex (8%). On the other hand, the NS2B (48–100;Δ77–84)-NS3pro complex contains much lower strand (8%) but higher (36%) turn conformations than the isolated NS3pro and NS2B (48–100)-NS3pro complex. Interestingly, the NS2B (48–100;Δ77–84)-NS3pro complex has ~37% unstructured conformation, slightly higher than that of the NS2B (48–100)-NS3pro complex (31%), but much lower than that of the isolated NS3pro (53%). These results indicate that the NS2B (48–100;Δ77–84)-NS3pro complex has a very different structure from either isolated NS3pro or NS2B (48–100)-NS3pro complex.

Indeed, very different from the HSQC spectra of the isolated NS3pro (Fig 1B) or NS2B (48–100)-NS3pro complex (Fig 4D), only a small set of HSQC peaks could be detected for the ^15^N-labeled NS3pro in complex with NS2B (48–100;Δ77–84) (Fig 8B). Furthermore, a large portion of detectable HSQC peaks are superimposable to those of the NS2B (48–100)-NS3pro complex (Fig 8B), implying that some residues of the NS2B (48–100;Δ77–84)-NS3pro complex have conformations similar to those of the NS2B (48–100)-NS3pro complex. However, HSQC peaks of the majority of residues were not detected, indicating that the NS2B (48–100;Δ77–84)-NS3pro complex undergoes conformational exchanges on μs-ms, or/and dynamic oligomerization, thus retarding further NMR characterization of its high-resolution conformation. Most strikingly, the NS2B (48–100;Δ77–84)-NS3pro complex had no detectable catalytic activity even with a concentration up to 10 μM in buffers at pH 7.5, suggesting that this complex is trapped in a highly-dynamic and catalytically impotent state.

## Discussion

As virus-encoded proteases have been shown to be essential for the replication and infectivity of many viruses, consequently they become important targets for design of anti-viral drugs [42,43]. Indeed, drugs have been successfully developed to treat HIV and HCV infections by targeting their proteases [44,45]. The Dengue NS3 protease has its catalytic machinery hosted by a chymotrypsin fold, which has been extensively shared by a variety of proteases. On the other hand, unlike most other chymotrypsin-like proteases, only flavivirus proteases including dengue NS3 protease need an additional cofactor NS2B to form the enzymatically active complex. Noticeably, even within the flavivirus proteases, the NS2B cofactors are very divergent, only with sequence identity of ~19% among different members [6]. So the NS2B-NS3pro interface may represent an attractive target for developing molecules which specifically inhibit flavivirus proteases. However, due to its “complete insolubility”, the isolated NS3pro domain has never been experimentally characterized so far.

In the present study, as facilitated by our discovery in 2005 [15–19], for the first time, the isolated NS3pro domain has been extensively characterized in solution by CD, NMR and PRE. The results decipher an unexpected fact that despite owning a high-complexity sequence which is very different from classic IUPs [31–36], the NS3pro with the native sequence is intrinsically disordered without the NS2B cofactor, with no stable secondary structures and tight tertiary packing. This indicates that the isolated NS3pro becomes completely insoluble in buffers by following the same mechanism as we previously established for other “completely insoluble” proteins [15–19]. Nevertheless, in the presence of the NS2B cofactor, the disordered NS3pro domain folds into the well-structured chymotrypsin-like fold hosting the active catalytic machinery by the “binding-coupled folding” mechanism [32–36]. Previously, the chymotrypsin fold has been found to be an autonomous folding unit even in the context of the SARS 3C-like protease with an extra domain [46]. Therefore, to the best of our knowledge, the NS3pro domain represents the first intrinsically-disordered chymotrypsin-like fold which absolutely requests the additional cofactor to achieve its correct folding. So an interesting question is whether being intrinsically disordered for the isolated NS3pro bears any *in vivo* relevance? Interestingly, a recent study has revealed that the dengue NS3pro domain without NS2B was capable of binding human fatty acid synthase (FASN) to trigger the membrane remodelling [47]. As such, our biophysical results imply that this interaction needs the dengue NS3pro to be largely disordered. Indeed, we found that the well-folded NS2B-NS3pro complex only had weak binding to the FASN domain. Furthermore, DENV and other members of the *Flaviviridae* family are dependent on the host ER to translate, replicate and package their genome, and their infection has been found to induce significant rearrangements of intracellular membranes [47–50]. In particular it has been recently revealed that the rearrangement and expansion of the ER appear to be driven by viral but not host protein synthesis early after DENV infection, independently of the UPR or SREBP2 pathways [50]. Therefore, on a speculative note, we propose that the highly disordered NS3pro itself might also play a role in triggering the early rearrangement of the ER before activating specific pathways [50]. Previously, we have shown that the ALS-causing P56S mutation of the ER-anchored VAPB protein rendered the well-structured β-barrel fold of its MSP domain to be highly disordered and also become “completely insoluble” in buffer [51], similar to what we found here on the isolated NS3pro. Remarkably, the P56S-VAPB suddenly gained the novel capacity to remodel the ER structure which was not observed for the wild-type VAPB [52]. Therefore, the disordered human P56S-VAPB and dengue NS3pro may use similar mechanisms to remodel the ER structure. In fact, the degue NS3 protein itself is also anchored into the ER membrane before NS3 forms the catalytically-active complex with NS2B to cleave itself from the polyprotein.

In this study, with our previous discovery that “insoluble proteins could be solubilized in water with minimized salt ions, we also successfully refolded the NS3pro protease with the full-length NS2B (1–130) anchored into the LMPC micelle. Interestingly, although NMR characterization deciphers that the NS3pro domains have different dynamics on the μs-ms time scale in the contexts of being complexed with NS (48–100) in buffer and with NS2B (1–130) in the LMPC micelle, they have very similar enzymatic activities. This membrane-anchored complex may provide a platform for screening the inhibitors for the dengue protease. In this regard, despite challenging, it is of both fundamental and therapeutic interest in the future to explore how the different structures and dynamics upon being anchored into membranes affect the affinity and specificity of the dengue NS3pro in binding substrates and inhibitors.

Further MD simulations provide critical insights into the indispensable role of the NS2B cofactor. Even within 20-ns simulations, the isolated NS3pro domain already showed higher dynamic instability than the NS3pro domain in the NS2B-NS3pro complex (Fig 5F). It is thus expected that on the long time scale, the isolated NS3pro would become unfolded as experimentally demonstrated here. Strikingly, the NS2B residues show much higher structural dynamics than NS3pro even in the complex, suggesting that the cofactor is capable of sampling a large ensemble of conformations, thus providing the dynamic mechanism for the observed conformational exchange of NS2B between open and closed conformations [8–12].

The correlated motions in proteins have been extensively recognized to be critical for their diverse functions [20,21,30,53]. In particular, we have recently revealed that a global correlated motion network was essential for the catalysis of the SARS 3C-like protease [20,21], which also utilizes the chymotrypsin fold to harbour its catalytic machinery. More specifically, without altering the three-dimensional structure of the enzyme, the N214A mutation on the extra domain is sufficient to inactivate the catalytic machinery by globally decoupling the correlation network, while the STI/A mutations also on the extra domain enhance the catalytic machinery by altering the correlation network pattern [20,21]. In the present study, a similar scenario has been observed for the dengue NS2B-NS3pro complex. A global correlation network does exist in the NS2B-NS3 complex. Most intriguingly, this global correlation network is mostly coordinated by the NS2B residues which have very high structural dynamics. As such, the MD results not only rationalizes the central role of the NS2B cofactor in maintaining the dynamic stability of the NS3pro domain, but further implies that a slight perturbation of the NS2B-NS3pro interface may be sufficient to decouple the correlation network to inactive the catalytic machinery of the dengue NS2B-NS3pro protease, as we found on the N214A mutation of the SARS 3C-like protease [20,21]. Indeed, by deleting the NS2B residues highly flexible in MD simulations, we obtained a truncated NS2B (48–100;Δ77–84), which is able to trap the NS2B-NS3pro complex in a catalytically-impotent state with significant μs-ms dynamics. In the further, it would be of significant interest to test whether this truncated NS2B can act as a specific inhibitor of the dengue protease *in vivo*.

Despite the success in treating viral infections with inhibitors to target the active sites of HIV and HCV proteases [41–43], many challenges/difficulties still remain for this approach. For example, the structural architecture and catalytic mechanism of viral proteases are also largely shared by many human proteases. This makes it extremely challenging to design inhibitors which only specifically bind to viral proteases but not to human ones. Moreover, there is an addition challenge associated with the dengue NS3pro protease. It has been proposed that the flat and charged nature of its active site may at least partly contribute to the current failure in developing effective inhibitors [7,45]. To overcome these difficulties, alternative strategies are requested to targets sites other than the active sites of the viral proteases, such as to inhibit the dimerization required for activity [46], to trigger allosteric inhibition [45], or even to block the folding of the protease [54]. In this regard, our present study successfully implies potential strategies to perturb the NS2B-NS3pro interface for future developing effective and specific inhibitors for the dengue protease. More specifically, inhibitory molecules might be developed to: 1) trap the NS2B-mediated folding into an inactive intermediate as exemplified by the truncated NS2B; and 2) decouple the global correlation network of the NS2B-NS3pro complex. Furthermore, our success in *in vitro* refolding of the active NS3pro in complex with both NS2B (48–100) in buffer and NS2B (1–130) in the micelle provides experimental platforms for implementing the above-proposed approaches of inhibitor design as well as to decode the underlying mechanism for inhibitors obtained by high through-put screening. For example, recently the dengue protease inhibitors were identified by high through-put screening, which were implied to target the NS2B-NS3pro interface by *in silico* prediction or mutagenesis [55,56]. It would be of significant interest to delineate how these molecules achieve the inhibitory effect: by trapping the folding or modulating the dynamics of the protease complex, if they directly target the NS2B-NS3pro interface.

In summary, our study decrypted that the dengue NS3pro domain is the first intrinsically-disordered chymotrypsin-like fold which absolutely requests the NS2B cofactor to coordinate the correct folding as well as correlated motions of the NS2B-NS3pro complex to achieve its catalytic function. In light of a recent study [47], the disordered NS3pro is needed for interacting with the human host factor to initiate the membrane remodeling. Furthermore, our results also imply potential strategies to manipulate the NS2B-NS3pro interface for design of molecules in the future, which may effectively and specifically inhibit the protease activity for treating the dengue infection.

## Supporting Information

S1 Fig Selective labels with the paramagnetic MTSL probe. (A) The NS3pro structure showing three mutation locations (Q27C, E86C and S158C) for labelling with the MTSL probe. Overlay of two ^1^H-^15^N HSQC spectra of labeled Q27C (B), E86C (C) and S158C (D) in the paramagnetic state of the MTSL probe (red) and diamagnetic state after the MTSL probe was reduced (blue).
(TIF)
