# Supplementary figures and images for: NMR and MD Studies Reveal That the Isolated Dengue NS3 Protease Is an Intrinsically Disordered Chymotrypsin Fold Which Absolutely Requests NS2B for Correct Folding and Functional Dynamics
Source: PLoS One. 2015 Aug 10;10(8):e0134823. doi: 10.1371/journal.pone.0134823 (PMC4530887; doi:10.1371/journal.pone.0134823)

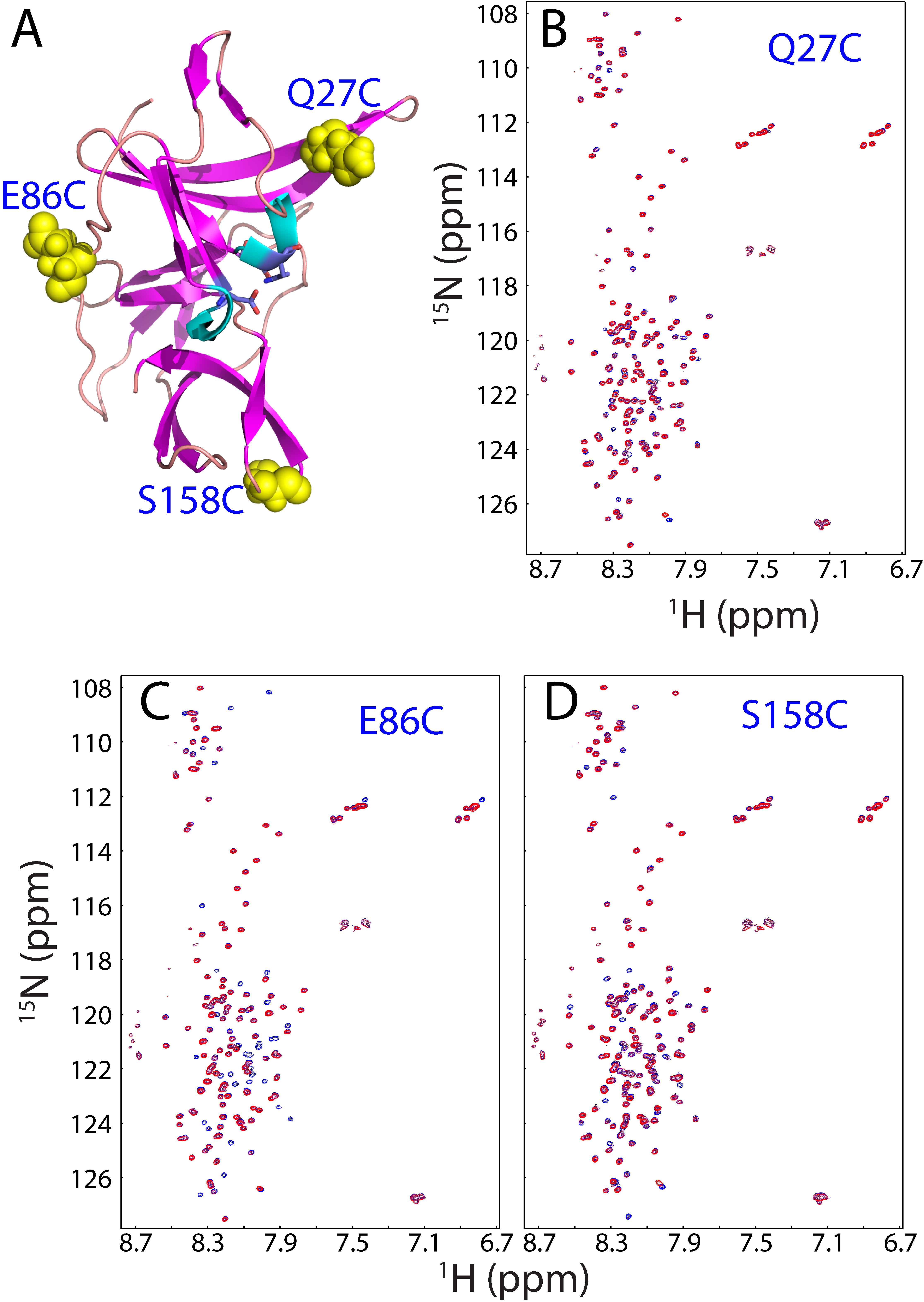

Supplement: S1 Fig — (A) The NS3pro structure showing three mutation locations (Q27C, E86C and S158C) for labelling with the MTSL probe. Overlay of two 1H-15N HSQC spectra of labeled Q27C (B), E86C (C) and S158C (D) in the paramagnetic state of the MTSL probe (red) and diamagnetic state after the MTSL probe was reduced (blue). (TIF) [file pone.0134823.s001.tif]
